# Does *Colostrum Bovinum* Supplementation Affect Swimming Performance in Endurance-Trained Males? A Randomized Placebo-Controlled Crossover Study

**DOI:** 10.3390/nu16183204

**Published:** 2024-09-22

**Authors:** Krzysztof Durkalec-Michalski, Natalia Główka, Tomasz Podgórski, Mikołaj Szymocha, Błażej Przybylik, Krystian Wochna, Małgorzata Woźniewicz, Paulina M. Nowaczyk

**Affiliations:** 1 Department of Sports Dietetics, Poznan University of Physical Education, 61-871 Poznań, Poland; glowka@awf.poznan.pl (N.G.); szymocha@awf.poznan.pl (M.S.); blazejprzybylik@gmail.com (B.P.); nowaczyk@awf.poznan.pl (P.M.N.); 2 Department of Physiology and Biochemistry, Poznan University of Physical Education, 61-871 Poznań, Poland; podgorski@awf.poznan.pl; 3 Department of Swimming and Water Lifesaving, Poznan University of Physical Education, 61-871 Poznań, Poland; kwochna@awf.poznan.pl; 4 Department of Human Nutrition and Dietetics, Poznań University of Life Sciences, 60-624 Poznań, Poland; malgorzata.wozniewicz@up.poznan.pl

**Keywords:** supplementation, sport nutrition, triathlon, swimming, ergogenic support

## Abstract

**Background/Objectives**: *Colostrum Bovinum* (COL) is recognized for its unique composition and potential ergogenic and immunological benefits. Unlike mature milk, COL is rich in immunoglobulins, lactoferrin, and various growth factors, making it one of the most potent natural immune stimulants. The purpose of this study was to evaluate the effects of 12-weeks of COL supplementation on swimming-specific performance (SSP) and exercise adaptations in endurance-trained male athletes. **Methods:** Twenty-eight male triathletes and swimmers (age: 31.1 ± 10.2 years; body mass: 81.9 ± 9.0 kg; height: 1.82 ± 0.06 m) participated in a randomized, double-blind, placebo (PLA)-controlled crossover study and received 25 g∙day^−1^ of COL or PLA for 12 weeks. The study assessed the effects of COL on SSP (8 × 100 m performed at various intensities) and exercise adaptations [heart rate (HR) and blood lactate concentrations ([*La^−^*])]. Four main study visits were conducted—before and after COL (*COL_PRE_* and *COL_POST_*) and PLA (*PLA_PRE_* and *PLA_POST_*) supplementation. **Results**: COL had no significant effect on SSP. Still, the total time of the SSP test was about ~3.04 s shorter after COL supplementation, and ~7.13 s longer after PLA supplementation. Neither COL nor PLA supplementation affected HR during the SSP test. Post-exercise blood [*La^−^*] was significantly reduced after both COL and PLA supplementation. The analysis of SSP results in the consecutive study visits revealed possible existence of the practice effect. **Conclusions**: *Colostrum Bovinum* and high-quality milk protein (PLA) seem to be comparably effective in evoking exercise adaptation in endurance-trained male athletes. Long-term crossover supplementation protocols in athletes must consider the impact of possible practice effect when interpreting the outcomes related to exercise performance, but not biochemical or physiological markers of exercise adaptation.

## 1. Introduction

For over 20 years, *Colostrum Bovinum* (COL) has been in the field of interest in sports nutrition research. Although its impact on physical capacity and recovery has been studied, and some research has shown its protective effects against decline in exercise capabilities due to intense training, there remains limited evidence regarding its influence on exercise performance [1].

COL is a substance secreted by the mammary glands of mammals during the first few days after giving birth (up to 48–72 h after calving). COL is characterized by a higher concentration of macronutrients (carbohydrates, proteins, and fats) [2,3] as well as biologically active substances [3] than human colostrum. The compounds contained in COL can be divided into three groups: nutrients, immune factors, and growth factors. The high concentration of bioactive components has led scientists to investigate the benefits of COL supplementation for exercise performance. However, potential effects on performance and recovery may be achieved through improved health outcomes, maintenance of immune functions, and protection of the intestines, as well as improved training adaptations [1].

Recent research on the impact of COL on physical performance has centered on three key areas: body composition and strength, high-intensity intermittent exercise, and endurance. Given that COL contains a higher concentration of micronutrients compared to whey, it may significantly contribute to adaptations during intense training [1]. Some studies [4,5] have shown that when combined with resistance training, COL supplementation may lead to increased muscle strength, muscle mass, and fat loss compared to a placebo. However, other research has found no significant differences [6,7]. These inconsistent results may be due to earlier studies using maltodextrin as a placebo, which does not have the same protein content as COL.

One of the aspects that COL studies focus on is the improvement in endurance performance, which may be achieved through enhanced recovery, buffering capacity and training adaptations [1]. In one such study, Kotsis et al. [8] showed that a low dose of COL (3.2 g_COL_∙day^−1^) over 6 weeks of supplementation reduced muscle damage (*C*-reactive protein, creatine kinase) and inflammatory (interleukin 6) markers after an exercise task simulating the physiological demands of soccer practice. This suggests COL may support performance adaptations. Moreover, Buckley et al. [9] found that a high dose (60 g_COL_∙day^−1^) of COL improved running performance, based on an incremental running test, after 8 weeks compared to a placebo (PLA), with greater improvements in distance covered. Additionally, Shing et al. [10] demonstrated that 10 g_COL_∙day^−1^ for 6 weeks prevented a decline in time-trial (TT) performance following a 5-day intensified training period and increased TT intensity (percentage of maximal oxygen uptake [VO_2MAX_]) while maintaining the ventilatory threshold. Furthermore, Coombes et al. [11] found that both 20 and 60 g_COL_∙day^−1^ for 8 weeks improved endurance performance in cyclists, as measured by cycling work capacity (a work-based TT following a 2-h cycle at 65% VO_2MAX_). 

Additionally, it is worth emphasizing that COL contains several growth factors, such as proteins, insulin-like growth factor-1 (IGF-1), or transforming growth factor-beta (TGF-β1), which may also indirectly affect performance through the stimulation of protein synthesis, the modulation of muscle growth and recovery, as well as the enhancement of immune function, gut health, and anti-inflammatory effects. A study by Buckley et al. [12] showed that athletes who took COL for 8 weeks experienced shorter recovery time and less muscle soreness compared to those who did not. The COL group also showed enhanced performance in sprint tests after recovery. Moreover, prolonged or intense exercise can suppress the immune system, leaving athletes more susceptible to illness. Meanwhile, COL contains immunoglobulins (IgG, IgA) and lactoferrin, which help support immune health by neutralizing pathogens and boosting immune responses. Jones et al. [13] found that athletes using COL had a lower incidence of upper respiratory tract infections, which is a common issue among endurance athletes, allowing them to train more consistently. Intense endurance exercises can lead to gastrointestinal permeability, which hampers nutrient absorption and leads to inflammation. COL contains growth factors (i.e., IGF-1, TGF-β1) that help repair and strengthen the gut lining. Shing et al. [14] found that COL supplementation in athletes helped maintain intestinal integrity during endurance events, which might prevent the performance drop linked to gut disfunctions and ensure better nutrient absorption for energy demands during prolonged exercise. COL’s anti-inflammatory properties are linked to compounds like lactoferrin and certain cytokines. These bioactive compounds reduce muscle and joint inflammation caused by exercise, which aids in faster recovery and less discomfort. Inflammation is a natural part of the recovery process, but excessive or prolonged inflammation can slow down muscle repair. COL helps moderate inflammation to ensure it is controlled without hampering recovery [15].

While some studies have examined the effectiveness of COL supplementation on exercise performance, additional research is necessary. Future studies should incorporate well-matched PLA and control groups to thoroughly assess the impact of COL on endurance performance during athletic training.

Therefore, the objective of this study was to assess the impact of a 12-week regimen of 25 g_COL_∙day^−1^ of COL supplementation in a group of healthy, moderately endurance-trained males engaged in triathlon and swimming practice. We assessed the impact of COL supplementation on exercise performance and adaptations in the body to exercise based on selected physiological (heart rate; HR) and biochemical markers. We hypothesized that COL supplementation would improve results in the swimming-specific performance (SSP) test (8 × 100 m of swimming performed at various intensities)—namely, shortened times for particular distances and the total times for SSP test overall (primary outcomes). We also hypothesized that COL supplementation would trigger favorable changes in exercise adaptation, evaluated based on HR and blood lactate concentrations ([*La^−^*]; secondary outcomes). 

## 2. Materials and Methods

### 2.1. Study Participants 

**Figure 1:**
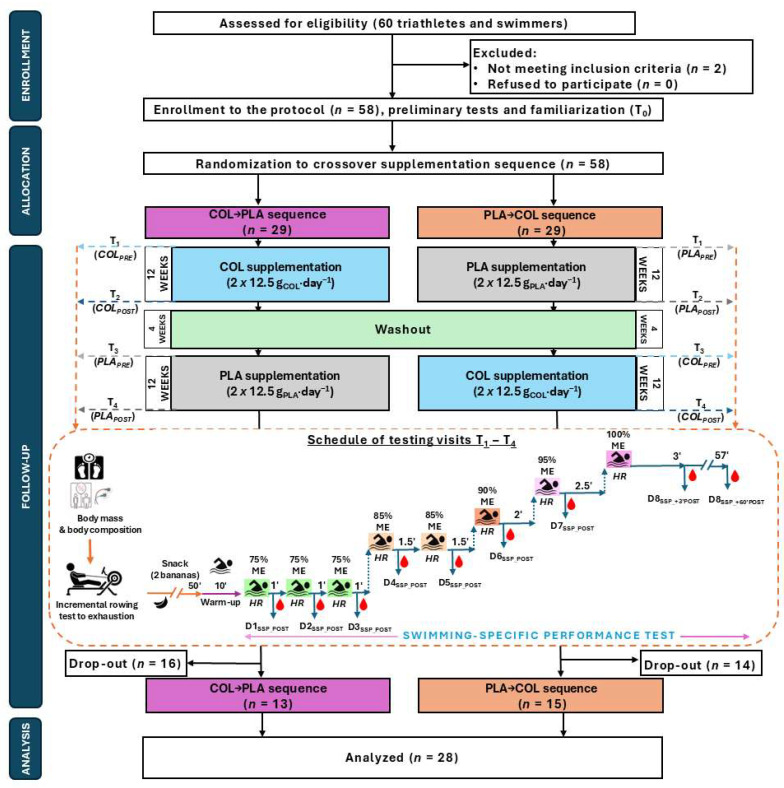
Flowchart of the study protocol. Abbreviations: COL, *Colostrum Bovinum*; D_SSP_, the distance number in swimming-specific performance test; HR, heart rate; ME, maximal effort; PLA, placebo; SSP, swimming-specific performance test.

The study initially enrolled 58 male participants involved in moderate endurance training (at least 3–5 times per week). However, 30 participants dropped out due to various reasons: injuries (*n* = 8), antibiotic therapy (*n* = 4), business trips (*n* = 3), family reasons (*n* = 3), and withdrawing without providing a reason during the washout period (*n* = 12). Ultimately, 28 athletes (31.1 ± 10.2 years; 81.9 ± 9.0 kg body mass (BM); 1.82 ± 0.06 m height; 68.5 ± 6.2 kg fat-free mass [FFM]; 13.3 ± 5.5 kg fat mass [FM]), including 17 triathletes and 11 swimmers, completed the entire study protocol (see Figure 1). The participants were recruited primarily from sports clubs in Poland (mainly Poznań, Szczecin, and Wrocław). 

Eligibility criteria required participants to be healthy, to not have chronic and/or autoimmune diseases, and to have a valid medical certificate to prove their fitness for sports. Alongside these criteria, participants needed to have been engaged in triathlon or swimming for at least 5 years and to have regularly participated in national competitions (at least 2–3 times per year). The participants in our study could be categorized as being at least Tier 2 (trained/developmental) according to the latest training status classification framework by McKay et al. [16]. Exclusion criteria included being allergic to cow‘s milk proteins, being lactose intolerant, showing symptoms of infection or use of any drugs within the last month prior to the study. All athletes claimed that they did not change their way of life, training process, eating habits, or supplementation throughout the research.

The Bioethics Committee at the Poznan University of Medical Sciences (identifier: 486/19) approved the research protocol and the study was registered at ClinicalTrials.gov (NCT06390670). This study followed CONSORT guidelines and is in line with the ethical aspects of the Declarations of Helsinki 2013. With the use of G*Power software version 3.1.9.4 (Universität Düsseldorf, Germany), sample size calculations were done to ensure approximately an 80% power (α = 0.05) and partial eta squared large effect size 0.14 under repeated measures with the ANOVA framework. The analysis revealed that a convenient sample size for this study was 26 participants.

### 2.2. Study Design and Visits

A randomized double-blind crossover design was utilized in this study, which followed a 12-week supplementation protocol with either COL or PLA. The protocol included an initial familiarization session (T_0_) and four main laboratory visits (T_1_–T_4_) carried out before and after the supplementation periods (*COL_PRE_*, *COL_POST_*, *PLA_PRE_*, and *PLA_POST_*). Visits T_1_ and T_3_ served as baseline assessments before supplementation (see Figure 1). The research was conducted in several waves between November 2021 and May 2023 at the Poznan University of Physical Education in Poland. Each wave commenced either during autumn or winter and finished before spring or summer to balance the risks associated with upper respiratory tract infection occurrences.

After familiarization, participants were randomly assigned into supplementation sequences groups (COL→PLA or PLA→COL) via stratified randomization based on body composition results, handled by an impartial biostatistician. A 4-week washout period was included between the treatment phases. Each visit (T_1_–T_4_) involved evaluations of BM and body composition, with nine blood samples taken in total: during the SSP test and 3 and 60 min post-exercise (see Figure 1). 

Testing was consistently scheduled in the morning to ensure standardization of study conditions and to minimize the effects of diurnal physiological fluctuations in the obtained results. Participants consumed a standardized meal three hours before each visit [17,18,19].

#### 2.2.1. Supplementation

In the experimental procedure, each athlete was given a daily dose of 25 g of either COL or PLA over 12 weeks, following a randomized crossover sequence. The COL supplement, derived from the first post-delivery milking, contained a high immunoglobulin G content (30%; certified *Colostrum Bovinum*; Agrapak, Poznań, Poland). The PLA was an isoenergetic and isomacronutrient product made from high-quality milk protein (Agrapak, Poznań, Poland). Both supplements were in powder form and taken twice daily (12.5 g in the morning and 12.5 g in the afternoon), dissolved in 250 mL of plain water. 

To ensure blinding, unique codes marked all containers that distributed the supplements. A researcher who was not directly involved in conducting research procedures and evaluating results was responsible for the preparation of the supplements (COL and PLA) beforehand. Thus double-blind settings were maintained, and neither participants nor researchers knew whether the participant was receiving COL or PLA at the particular supplementation phase, according to procedures of the highest standards of good practices in conducting clinical trials [20]. Until the final completion of this study, randomization details were anonymized. Out of 28 participants who completed the entire study protocol, as many as 12 athletes declared that they were sure of what supplement they had taken during each phase; but only 2 participants were in fact right about the sequence of supplementation. This fact confirms a full effectiveness of the implemented double-blinding procedure.

#### 2.2.2. Body Mass and Body Composition Evaluation

Participants were asked to refrain from engaging in any form of exercise 24 h prior to any visit. At the beginning of each visit, the anthropometric measurements were taken to keep the test conditions as equal as possible. The BM and height were measured with a calibrated scale with a stadiometer (WPT 60/150 OW, Radwag, Radom, Poland). Body composition was analyzed using electrical bioimpedance with a BIA-101ASE device (Akern, Pontassieve, Italy), following all recommended procedures for measurement conditions as previously described [21]. This analysis determined total body water (TBW; L and %), FFM (kg and %), and FM (kg and %). 

#### 2.2.3. Exercise Protocol

During the SSP tests in the indoor 25 m lap swimming pool, all recommended conditions for such assessments were kept constant and aligned with the recommendations set out by World Aquatics (previously known as the *Fédération Internationale de Natation* [*FINA*]) [22,23]. The ambient air temperature was maintained between 30–34 °C, whereas the water temperature was not less than 28 °C, with a relative humidity of around 60–70%. These tests were performed at the same time of day for all the participants (in the morning). Screening was done at the time of enrolment (T_0_) where the participant was tested on the procedures before going through the main procedure (T_1_–T_4_) as described in the protocol.

The test was performed after a 10-min warm-up in the swimming pool. The SSP test comprised of performing eight 100-m distances split into ramp parts with increasing intensity. Ramps one to three (D1_SSP_, D2_SSP_, D3_SSP_) were swum at 75% of the individual’s maximal effort (ME; assessed one week before each study visit); ramps four (D4_SSP_) and five (D5_SSP_) at 85% ME; ramp six (D6_SSP_) at 90% ME; ramp seven (D7_SSP_) at 95% ME; and ramp eight (D8_SSP_) at 100% ME. Recovery periods between each ramp ranged between 1 and 2.5 min, as shown in Figure 1. The step test we implemented is a modified version of a previously validated swimming lactate threshold protocol [24,25]. During the SSP test, the time for each 100-m section, as well as the total time for the whole SSP test, was measured using a stopwatch (Garmin Fēnix 5X, Garmin, Olathe, KS, USA); for accuracy, the time for each test was measured to a hundredth of a second. 

Moreover, HR was constantly monitored using a telemetric system (Garmin Fēnix 5X and HRM-Swim™; Garmin, Olathe, KS, USA) during the SSP test. From the collected data, HR_MEAN_ and HR_MAX_ were determined. 

#### 2.2.4. Blood Collection and Sample Analysis

Fingertip capillary blood samples were collected at nine different time points during each study visit: seven times during exercise protocol (immediately after each set of 100-m section, during the recovery time after each section—D1_SSP_POST_, D2_SSP_POST_, D3_SSP_POST_, D4_SSP_POST_, D5_SSP_POST_, D6_SSP_POST_, and D7_SSP_POST_,) as well as 3 (D8_SSP_+3’POST_), and 60 min (D8_SSP_+60’POST_) after completion of D8_SSP_ (see Figure 1). Each blood sample (50 μL) was promptly placed into microtubes containing 250 μL of 0.6 M perchloric acid. An [*La^−^*] measurement was performed according to the method described previously by Maughan [26], and successfully implemented in our earlier studies [17,27].

### 2.3. Statistical Analysis

Variables were checked for a normal distribution with the Shapiro–Wilk test. Furthermore, kurtosis, skewness [28], and a graphical evaluation of the distribution of each variable data were performed. Data was analyzed with analysis of variance with repeated measurements (RM ANOVA), with the effect size (ES) expressed as partial eta-squared (*η*^2^*_p_*). A Huynh–Feldt adjustment was made when sphericity was violated (as indicated by Mauchly’s test). Post-hoc comparisons were performed using the Bonferroni test for multiple comparisons. The ES was interpreted as follows: *η*^2^*_p_*: <0.010 *no* effect; from 0.010 to 0.059 *small* effect; from 0.060 to 0.139 *moderate* effect; and ≥0.140 *large* effect. To verify the possible practice effect, the comparisons in time of particular SSP distances and total SSP time between consecutive study visits (T_1_–T_4_) were performed using RM ANOVA. Moreover, differences in percentage changes in the times of the SSP tests between COL and PLA supplementation were analyzed using a *t*-test for dependent variables. An alpha of <0.05 was taken as a statistically significant value. The data were analyzed using the STATISTICA 13.3 software (StatSoft Polska Sp. z o.o., 2024, Zestaw Plus version 5.0.96, Poland).

## 3. Results

### 3.1. Swimming-Specific Performance

Neither COL nor PLA supplementation affected the total time of the SSP test or times at particular distances during the SSP test (D1_SSP_–D8_SSP_; Table 1). Nevertheless, the total time of the SSP test after COL supplementation was ~3.04 s shorter, while after PLA supplementation it was ~7.13 s longer compared to pre-supplementation measurements (Table 1).

**Figure 2:**
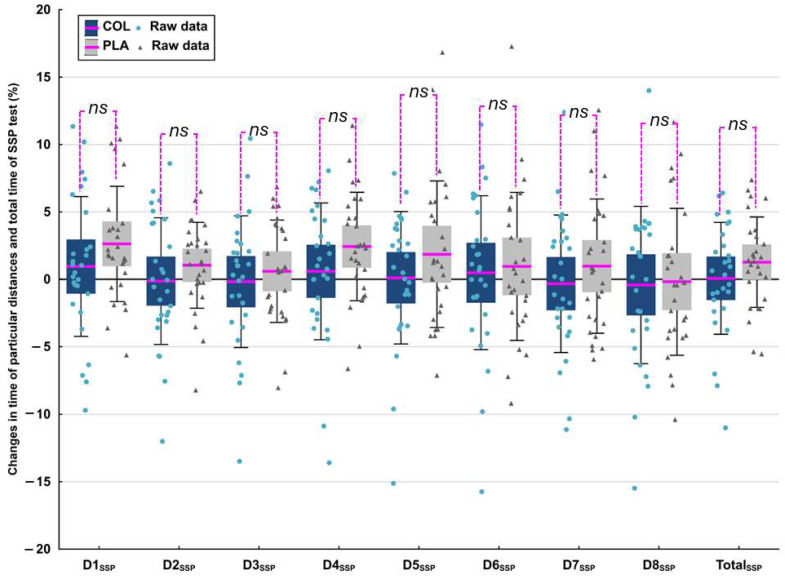
Changes in time (%) of particular distances in the swimming-specific performance (SSP) test and in total time of the SSP test. The data are expressed as the mean (line), 95% confidence interval (box), 95% CI + one standard deviation (whisker), and raw data of individuals. The data were analyzed with *t*-test for dependent variables. Abbreviations: COL, *Colostrum Bovinum*; D_SSP_, the distance number in swimming-specific performance test; *ns*, statistically not significant; PLA, placebo; SSP, swimming-specific performance test.

There were no significant differences in the percentage changes in time for particular distances or the total time of the SSP test between COL and PLA supplementation (see Figure 2).

### 3.2. Heart Rate during the SSP Test

COL and PLA supplementation had no effect on the HR_MEAN_. However, the HR_MEAN_ was significantly lower at *PLA_POST_* compared to *COL_PRE_*, with no differences between the remaining measuring time points (*p* = 0.011, *η*^2^*_p_* = 0.127; *COL_PRE_* vs. *PLA_POST_*: *p* = 0.006; see Table 2). COL and PLA supplementation had no effect on HR_MAX_ during the SSP test (see Table 2).

### 3.3. Blood Lactate Concentration

From D1_SSP_POST_ to D4_SSP_POST_ there were no differences in blood [*La^−^*] between measuring time points (see Table 3). At D5_SSP___POST_ (*p* < 0.001, *η*^2^*_p_* = 0.236; *COL_PRE_* vs. *PLA_POST_*: *p* = 0.001; *PLA_PRE_* vs. *PLA_POST_*: *p* < 0.001; Table 3), and D6_SSP___POST_ (*p* = 0.003, *η*^2^*_p_* = 0.176; *COL_PRE_* vs. *PLA_POST_*: *p* = 0.013; *PLA_PRE_* vs. *PLA_POST_*: *p* = 0.003) blood [*La^−^*] was significantly lower at *PLA_POST_* compared to *COL_PRE_* and *PLA_PRE_*, but with no differences between *COL_POST_* and the other measuring time points. At D7_SSP_POST_ (*p* = 0.001, *η*^2^*_p_* = 0.188; *COL_POST_* vs. *PLA_PRE_*: *p* = 0.029; *PLA_POST_* vs. *PLA_PRE_*: *p* = 0.001; Table 3), blood [*La^−^*] was significantly lower at *COL_POST_* and *PLA_POST_* compared to *PLA_PRE_,* with no differences between *COL_POST_* and *PLA_POST_*, as well as between *COL_PRE_* and remaining measuring time points. At D8_SSP_+3’POST_ (*p* < 0.001, *η*^2^*_p_* = 0.279; *COL_PRE_* vs. *COL_POST_*: *p* = 0.003; *PLA_PRE_* vs. *PLA_POST_*: *p* < 0.001; *PLA_PRE_* vs. *COL_POST_*: *p* < 0.001; *COL_PRE_* vs. *PLA_POST_*: *p* = 0.004; Table 3), blood [*La^−^*] was significantly lower at *COL_POST_* and *PLA_POST_* compared to *COL_PRE_* and *PLA_PRE_*, respectively (with no differences between *COL_POST_* vs. *PLA_POST_*). Moreover, [*La^−^*] was lower at *COL_POST_* vs. *PLA_PRE_*, and at *PLA_POST_* vs. *COL_PRE_*. At D8_SSP+60’POST_, no differences in blood [*La^−^*] between any measuring time points were found (see Table 3).

### 3.4. Practice Effect

The D1_SSP_ time (*p* = 0.005, *η*^2^*_p_*= 0.145; T_1_ vs. T_4_: *p* = 0.003; Table 4) was significantly shorter at the T_1_ compared to the T_4_, with no differences between the remaining study visits. The D8_SSP_ time (*p* = 0.009, *η*^2^*_p_* = 0.132; T_1_ vs. T_3_: *p* = 0.007; Table 4) was significantly shorter at the T_3_ compared to the T_1_, with no differences between the remaining study visits. There were no significant differences in the time for remaining distances, including the total time of the SSP test, between consecutive study visits (see Table 4).

## 4. Discussion

The present double-blind randomized placebo-controlled crossover study aimed to investigate the ability of a 12-week 25 g_COL_∙day^−1^ supplementation to enhance the swimming performance of endurance-trained male athletes. The outcomes of the study demonstrated that COL supplementation may not improve swimming-specific performance in triathletes and swimmers more than PLA. Moreover, similar effects of COL and PLA supplementation on adaptations in the body to exercise based on selected physiological (HR) or biochemical ([*La^−^*]) markers were observed.

To the best of our knowledge, this is the first study conducted on swimmers and triathletes in terms of the effect of COL supplementation on swimming performance. Only one previous study [29] examined COL effectiveness in swimmers, but no exercise protocols were implemented and so there was no assessment of COL’s influence on performance. In our current study, exercise performance measured by the SSP test showed no differences between COL and PLA in the total time of the SSP test, or the time for the particular distances during the SSP test performed at various intensities. Nevertheless, after COL supplementation, the participants finished the test faster than after PLA. In terms of endurance performance, there is evidence suggesting that COL supplementation may enhance recovery from various types of strenuous exercise and improve subsequent performance [1]. Additionally, COL has been demonstrated to enhance recovery from repeated exercise sessions [12], to improve TT performance after extended submaximal exercise [11], and to sustain exercise performance after a period of high-intensity training (HIT) [10]. Buckley et al. [12] conducted a study where 39 male participants were given either COL (60 g∙day^−1^) or PLA over an 8-week period. During this time, their training included three 45-min running sessions per week. The participants underwent two incremental treadmill tests, separated by a 20-min passive recovery, at the start of the study, and again after 4 and 8 weeks of supplementation. After 4 weeks, no significant changes in running performance were observed. However, after 8 weeks, those in the COL group covered a significantly greater distance and completed more work in the second treadmill test compared to the PLA group (a 4.6% increase; *p* < 0.04). For these reasons, the remarkable enhancement of running performance observed could not be explained by any increases in respiratory exchange ratio, lactate threshold, or even insulin-like growth factor (IGF-1) concentrations. Also, on COL supplementation, no effect on VO_2MAX_ was observed, though there was improved endurance performance [11,12]. In a double-blind, placebo-controlled study, performed by Coombes et al. [11], 42 cyclists underwent a cycle TT of 2.8 kJ∙kg^−1^ after a 2-h endurance ride, both before and after an 8-week supplementation period. Participants were divided into groups receiving either 20 or 60 g_COL_∙day^−1^ or a PLA (whey protein powder). Results revealed a significant improvement in TT performance for those who took COL compared to PLA, with time decreases of 158 s in the 20 g_COL_∙day^−1^ group and 134 s in the 60 g_COL_∙day^−1^ group (both *p* < 0.05). The comparable performance gains in both COL groups suggest that increasing COL dose beyond a certain point may not yield additional performance benefits [1,30]. Although, improvements in endurance performance after COL supplementation do not seem to be linked to increased circulating IGF-1 levels, a study by Shing et al. [10] demonstrated that COL maintained ventilatory threshold and enhanced economy during periods of HIT. A dose of 10 g_COL_∙day^−1^ improved 40 km TT (TT40) performance following a 5-day HIT regimen, but not during regular training, compared to a whey protein PLA [10]. It appears that although the effects of COL on performance remain unclear, COL supplementation may be particularly advantageous for endurance performance during intense training phases or periods of overload, which might lead to fatigue and a reduced ventilatory threshold [1,30]. 

Adaptation of the body to exercise can be measured based on selected physiological (HR) and biochemical ([*La^−^*]) markers. Training adaptations based on these markers refer to the physiological changes that occur as a result of consistent exercise. In sports practice, HR and [*La^−^*] should be indicated as the most important and clinically relevant markers for monitoring the intensity and specificity of energy processes during exercise (aerobic vs. anaerobic). In our study, there were no differences in HR_MEAN_ or HR_MAX_ during the SSP test between COL and PLA. HR, reflecting the body’s ability to supply oxygen to working muscles during prolonged exercise, serves as an important indicator of endurance performance. A lower resting HR and quicker recovery after exercise also signal improved cardiovascular endurance. HR monitoring enables athletes to regulate the intensity of their training to achieve the desired improvement of their endurance-related capabilities as well as performance [31]. The majority of studies [12,32,33,34,35,36,37] that have examined HR during exercise, similarly to our results, showed no differences in HR (peak or mean) between COL and PLA treatments. Nevertheless, in one of the previously described studies, Shing et al. [10] showed that at the end of the HIT period, compared to PLA, COL maintained TT40 HR (2.5 ± 3.7%; a possible benefit to enhance TT40 intensity and prevent a decrease in submaximal HR during high-intensity training).

In the current study, there were no differences between COL and PLA in [*La^−^*] during/after the SSP and after 60 min of recovery. The lactate threshold refers to the point during exercise at which lactate—a byproduct of anaerobic metabolism—begins to accumulate in the bloodstream at a faster rate than it can be removed. This threshold is an important marker for endurance athletes, as it represents the intensity of exercise at which the body transitions from primarily aerobic energy production to increased reliance on anaerobic pathways [38]. In terms of high-intensity and intermittent exercise performance, some research [6,8,39] has suggested that COL might improve this type of performance by either increasing the intracellular buffer capacity to neutralize hydrogen ions (H^+^) or boosting the extracellular buffer capacity to allow greater H^+^ efflux from the muscles. Similarly to our results, no differences in [*La^−^*] were found in other studies [12,34,35,37,39,40]. Interestingly, Brinkworth et al. [39] proposed that COL supplementation might reduce the rate of intramuscular acidosis during intense exercise. The impact of COL on blood buffering capacity was examined during a 9-week training program involving 13 elite female rowers, who were given either 60 g∙day^−1^ of COL or 60 g∙day^−1^ of whey protein. The study utilized two incremental rowing tests (each consisting of four 3-min stages and separated by 15 min) to assess performance before and after the supplementation period. After 9 weeks of COL supplementation blood buffering capacity was increased based on the evaluation of blood [*La^−^*] and pH at the end of each workload during the tests. Despite the increased blood buffering capacity, no significant differences in exercise performance were found between the groups. In a follow-up study, Brinkworth and Buckley [6] reanalyzed the data from previous work [39] to determine which component of blood buffering capacity was enhanced by COL. The analysis revealed no significant differences in resting hemoglobin levels, plasma bicarbonate concentrations, or plasma buffering capacity (all systemic buffers) between the groups. The observed increase in blood buffering capacity may have resulted from either an increase in intracellular phosphate levels [30] or an improvement in hemoglobin’s ability to buffer H^+^. In a different investigation, Kotsis et al. [8] investigated how recovery performance following an intermittent exercise program intended to replicate the physiological demands of soccer was affected by a 6-week course of low dose (3.2 g_COL_∙day^−1^) COL supplementation. These results showed that COL accelerated the recovery of explosive power, as assessed by squat jump performance, and decreased markers of inflammation and muscle damage brought on by the workout regimen. These impacts may have a significant influence on training for intermittent sports adaptations and long-term performance gains. Further research is needed to determine if COL supplementation increases muscle buffering capacity, which would require direct measurement of muscle pH and [*La^−^*]. On the other hand, it must be emphasized that in the current study, COL supplementation resulted in significantly decreased [*La^−^*] (post- vs. pre-supplementation) at D8_SSP_+3’POST_, while PLA supplementation significantly decreased [*La^−^*] (post- vs. pre-supplementation) at D5_SSP_POST_, D6_SSP_POST_, D7_SSP_POST_, and D8_SSP_+3’POST_. These observations suggest improved bodies adaptation to exercise after COL and PLA (high-quality milk protein) supplementation. In this respect, lower [*La^−^*] (during- and post-exercise) after COL and PLA interventions and similar/improved exercise performance capabilities (a clinically significant shorter total time for the SSP test after COL) may be evidence of improved aerobic contribution to physical effort, better energetic economy, lower physiological load on the body, and therefore better exercise adaptation.

To summarize, COL has been marketed as a potential supplement for improving performance in endurance sports due to its high concentration of growth factors, immunoglobulins, and bioactive compounds. However, scientific evidence does not consistently support its effectiveness for enhancing endurance performance, which was shown in our current study. Endurance sport disciplines rely heavily on aerobic fitness and capacity, which is measured by VO_2max_. Studies have shown that COL supplementation does not lead to significant improvements in VO_2max_, which is a key factor in endurance performance. A study by Buckley et al. [12] demonstrated that although COL increased lean body mass, it had no significant effect on VO_2max_ or time to exhaustion in trained endurance athletes. Moreover, while COL is known to contain immunoglobulins that could potentially aid immune function, its impact on athletes’ immunity during prolonged exercise remains uncertain. Endurance athletes often suffer from immune suppression after intense training or competition, leading to illnesses that can hinder performance. Some studies showed mild improvements in immune markers, but these effects are not consistent across all research. A study by Jones et al. [41] found that COL might help maintain immune function, but these benefits do not translate into direct performance gains in endurance sports. Furthermore, for endurance athletes, maintaining efficient energy metabolism is also critical. COL does not appear to significantly enhance carbohydrate or fat oxidation, which are crucial for sustained endurance performance. Research published by Shing et al. [42] indicated that while COL might enhance recovery, its impact on energy metabolism during prolonged exercise remains unclear, limiting its usefulness for boosting endurance. In general, the results of studies examining COL supplementation and endurance performance are inconsistent. Some studies report minor improvements in recovery time or reduction in gut permeability (which can be beneficial during long-duration events), but these findings are equivocal. Still, the variation in study outcomes may be attributed to differences in study design, dosage, and duration of supplementation, making it difficult to draw definitive conclusions about the effectiveness of COL for endurance performance.

This study stands out for taking a novel strategy, especially as it uses a crossover design and supplementation with 25 g_COL_∙day^−1^ for 12 weeks. One of the crossover strategy’s main advantages is that it lessens the impact of variables and inter-individual variability in group comparisons. Future studies should assess the long-term effects of COL supplementation on athletes within regular training cycles, possibly by comparing results across consecutive seasons or by using a parallel group design, given the difficulty of accounting for changes in performance over an extended period of time. A carefully planned 4-week washout time in between treatments further helped our study since it addressed the intricacies of COL’s pharmacokinetics, which include bioactive components with variable elimination half-lives. A balanced comparison between seasons was ensured by having all participants start the study during the autumn/winter season. By keeping each participant’s visit timings consistent, the study protocol was further reinforced, which improved the reliability of the findings.

The study results could have been impacted by a number of limitations. Although a crossover design has its benefits, there are drawbacks as well, namely the possibility of carryover effects and the impact of treatment order on results. These issues are especially pertinent to research involving athletes, whose training loads change based on the unique cycles of their capabilities. Athletes are a demanding group to investigate, particularly when the additional supplementation intervention needs to avoid interfering with their training schedules. A further obstacle in the current study was the high dropout rate, which was mainly caused by participants quitting during the washout period (*n* = 12). This suggests that a parallel group design, which does not require a washout period, would have been a more appropriate and economical course of action. Injuries were the second most common reason for dropout (*n* = 8), underscoring the high risk of injury and illness among athletes, which can temporarily prevent them from training and competing. Despite these issues, the final sample size of 28 participants exceeded the a priori calculated requirement of 26, allowing the study to maintain its validity. Another possible limitation may have been the choice of the swimming protocol. Although we modified the original version of the 8 × 100 m SSP test to adjust it as well as possible to the capabilities of triathletes and swimmers, there is a possibility that a different swimming test would have shown different results. The chosen SSP test may have been too intense and too difficult for triathletes, possibly affecting their performance. Nevertheless, the main goal of this test was to induce as much fatigue as possible, and to evoke subsequent *La^−^* production as a response to high-intensity effort, which was clearly achieved. Based on changes in [*La^−^*] we were able to evaluate COL/PLA capabilities to provide exercise-induced adaptation.

## 5. Conclusions

In conclusion, 12 weeks of supplementation with 25 g_COL_∙day^−1^ in moderately-trained male triathletes and swimmers had no significant effect on swimming performance. Still, the total time of the swimming-specific performance test was about ~3.04 s shorter after COL supplementation, and ~7.13 s longer after PLA supplementation. Neither COL nor PLA supplementation affected heart rate during the specific exercise test. However, post-exercise blood lactate was significantly reduced after both COL and PLA supplementation. Long-term crossover supplementation protocols in athletes must consider the impact of a possible practice effect when interpreting the outcomes related to exercise performance, but not biochemical or physiological markers of exercise adaptation. *Colostrum Bovinum* and high-quality milk protein seem to be comparably effective in evoking exercise adaptation.

## Figures and Tables

**Table 1:** Time (min:s) in the swimming-specific performance test.

Distance	*COL_PRE_*	*COL_POST_*	*PLA_PRE_*	*PLA_POST_*	[*p*]; *η*^2^*_p_*
D1_SSP_ (75% ME)	1:30.51 ± 18.30 (1:23.42–1:37.61)	1:31.01 ± 17.02 (1:24.41–1:37.61)	1:30.18 ± 18.50 (1:23.01–1:37.35)	1:32.09 ± 16.80 (1:25.58–1:38.61)	[0.147]; 0.064
D2_SSP_ (75% ME)	1:32.06 ± 20.30 (1:24.19–1:39.93)	1:31.39 ± 17.79 (1:24.50–1:38.29)	1:31.53 ± 19.24 (1:24.07–1:38.99)	1:32.19 ± 18.07 (1:25.18–1:39.20)	[0.705]; 0.017
D3_SSP_ (75% ME)	1:32.38 ± 21.49 (1:24.05–1:40.72)	1:31.67 ± 18.92 (1:24.34–1:39.01)	1:32.36 ± 20.25 (1:24.51–1:40.21)	1:32.61 ± 19.10 (1:25.20–1:40.01)	[0.763]; 0.014
D4_SSP_ (85% ME)	1:29.42 ± 21.88 (1:20.93–1:37.91)	1:29.38 ± 19.46 (1:21.84–1:36.93)	1:28.77 ± 19.58 (1:21.18–1:36.36)	1:30.82 ± 19.93 (1:23.09–1:38.55)	[0.185]; 0.057
D5_SSP_ (85% ME)	1:27.55 ± 22.07 (1:18.99–1:36.11)	1:28.60 ± 19.41 (1:21.07–1:36.13)	1:28.39 ± 19.81 (1:20.70–1:36.07)	1:29.61 ± 19.52 (1:22.14–1:37.27)	[0.565]; 0.047
D6_SSP_ (90% ME)	1:27.55 ± 22.07 (1:18.99–1:36.11)	1:27.49 ± 20.15 (1:19.68–1:35.31)	1:26.95 ± 20.25 (1:19.09–1:34.80)	1:27.60 ± 20.26 (1:19.75–1:35.46)	[0.930]; 0.005
D7_SSP_ (95% ME)	1:26.18 ± 21.39 (1:17.88–1:34.47)	1:25.37 ± 19.17 (1:17.94–1:32.81)	1:25.62 ± 19.52 (1:18.05–1:33.19)	1:26.38–19.99 (1:18.63–1:34.13)	[0.678]; 0.018
D8_SSP_ (100% ME)	1:24.13 ± 21.18 (1:15.92–1:32.34)	1:23.27 ± 19.14 (1:15.85–1:30.70)	1:24.08–19.88 (1:16.38–1:31.79)	1:23.60 ± 18.85 (1:16.30–1:30.91)	[0.789]; 0.013
Total time of the SSP test	11:51.24 ± 2:45.69 (10:46.99–12:55.49)	11:48.20 ± 2:29.44 (10:50.26–12:46.15)	11:47.87 ± 2:35.89 (10:47.43–12:48.32)	11:55.01 ± 2:30.87 (10:56.51–12:53.51)	[0.586]; 0.023

The results are expressed as the mean ± standard deviation and 95% confidence interval (in parentheses). Abbreviations: D_SSP_, the distance number in swimming-specific performance test; ME, maximal effort; SSP, swimming-specific performance test. Data were analyzed with ANOVA with repeated measurements (RM ANOVA); the effect size expressed as partial eta square (*η*^2^*_p_*).

**Table 2:** Heart rate (bpm) during swimming-specific performance test.

Measured Indices	*COL_PRE_*	*COL_POST_*	*PLA_PRE_*	*PLA_POST_*	[*p*]; *η*^2^*_p_*
HR_MEAN_	143 ± 13 ^†^ (138–148)	139 ± 11 (135–143)	139 ± 12 (135–144)	135 ± 11 (131–139)	**[0.011]; 0.127**
HR_MAX_	175 ± 12 (170–180)	174 ± 13 (169–179)	173 ± 13 (168–179)	172 ± 11 (167–176)	[0.412]; 0.035

The results are expressed as the mean ± standard deviation and 95% confidence interval (in parentheses). Abbreviations: HR_MEAN_, mean heart rate during the swimming-specific test; HR_MAX_, maximal heart rate during the swimming-specific test. Data were analyzed with ANOVA with repeated measurements (RM ANOVA); the effect size expressed as partial eta square (*η*^2^*_p_*). Results in bold refer to statistically significant differences. ^†^ *COL_PRE_* vs. *PLA_POST_*: *p* = 0.006.

**Table 3:** Blood lactate concentration (mmol∙L^−1^).

Measuring Time Point	*COL_PRE_*	*COL_POST_*	*PLA_PRE_*	*PLA_POST_*	[*p*]; *η*^2^*_p_*
D1_SSP_POST_	5.0 ± 1.8 (4.4–5.8)	4.7 ± 1.9 (4.0–5.4)	5.3 ± 1.8 (4.6–6.0)	5.1 ± 1.8 (4.4–5.8)	[0.372]; 0.038
D2_SSP_POST_	6.1 ± 1.9 (5.3–6.9)	5.8 ± 2.5 (4.9–6.8)	6.3 ± 2.1 (5.5–7.1)	5.7 ± 1.7 (5.0–6.3)	[0.420]; 0.034
D3_SSP_POST_	6.9 ± 2.2 (6.0–7.7)	6.7 ± 2.6 (5.7–7.7)	6.8 ± 2.1 (6.0–7.6)	6.4 ± 2.0 (5.7–7.2)	[0.650]; 0.020
D4_SSP_POST_	7.8 ± 2.6 (6.8–8.8)	7.5 ± 2.5 (6.6–8.5)	7.9 ± 2.2 (7.0–8.7)	7.0 ± 2.0 (6.2–7.8)	[0.180]; 0.060
D5_SSP_POST_	8.7 ± 2.7 ^†^ (7.7–9.8)	7.8 ± 2.4 (6.8–8.7)	8.9 ± 2.2 ^‡^ (8.0–9.7)	6.8 ± 1.5 (6.2–7.4)	**[<0.001]; 0.236**
D6_SSP_POST_	9.7 ± 2.9 ^§^ (8.6–10.8)	8.6 ± 2.3 (7.7–9.5)	9.9 ± 2.0 * (9.2–10.7)	8.0 ± 1.6 (7.4–8.6)	**[0.003]; 0.176**
D7_SSP_POST_	10.5 ± 2.7 (9.5–11.6)	9.7 ± 2.8 ^††^ (8.6–10.7)	11.4 ± 2.3 (10.5–12.3)	9.0 ± 2.0 ^‡‡^ (8.2–9.8)	**[0.001]; 0.188**
D8_SSP_+3’POST_	13.0 ± 3.2 ^§§^ (11.7–14.2)	10.6 ± 3.5 ^~^ (9.2–11.9)	13.4 ± 3.5 ** (12.1–14.8)	10.6 ± 3.4 ^£^ (9.3–11.9)	**[<0.001]; 0.279**
D8_SSP_+60’POST_	2.2 ± 0.9 (1.9–2.6)	2.5 ± 1.3 (1.9–3.0)	2.6 ± 1.3 (2.1–3.1)	2.0 ± 0.7 (1.8–2.3)	[0.119]; 0.069

The results are expressed as the mean ± standard deviation and 95% confidence interval (in parentheses). Data were analyzed with ANOVA with repeated measurements (RM ANOVA); the effect size expressed as partial eta square (*η*^2^*_p_*). Results in bold refer to statistically significant differences. D5_SSP_POST_–^†^ *COL_PRE_* vs. *PLA_POST_*: *p* = 0.001; ^‡^ *PLA_PRE_* vs. *PLA_POST_*: *p* < 0.001. D6_SSP_POST_–^§^ *COL_PRE_* vs. *PLA_POST_*: *p* = 0.013; * *PLA_PRE_* vs. *PLA_POST_*: *p* = 0.003. D7_SSP_POST_–^††^ *COL_POST_* vs. *PLA_PRE_*: *p*= 0.029; ^‡‡^ *PLA_POST_* vs. *PLA_PRE_*: *p* = 0.001. D8_SSP_+3’POST_–^§§^ *COL_PRE_* vs. *COL_POST_*: *p* = 0.003; ** *PLA_PRE_* vs. *PLA_POST_*: *p* < 0.001; ^~^ *COL_POST_* vs. *PLA_PRE_*: *p* < 0.001; ^£^ *PLA_POST_* vs. *COL_PRE_*: *p* = 0.004.

**Table 4:** Time (min:s) in the swimming-specific performance test in the consecutive study visits.

Distance	T_1_	T_2_	T_3_	T_4_	[*p*]; *η*^2^*_p_*
D1_SSP_ (75% ME)	1:29.30 ± 17.66 ^†^ (1:22.45–1:36.15)	1:30.81 ± 17.52 (1:24.01–1:37.60)	1:31.39 ± 19.06 (1:24.00–1:38.78)	1:32.30 ± 16.26 (1:26.00–1:38.61)	**[0.005]; 0.145**
D2_SSP_ (75% ME)	1:31.73 ± 19.56 (1:24.14–1:39.32)	1:31.32 ± 18.29 (1:24.23–1:38.42)	1:31.86 ± 19.98 (1:24.11–1:39.61)	92.26 ± 17.56 (1:25.45–1:39.07)	[0.714]; 0.017
D3_SSP_ (75% ME)	1:32.78 ± 21.46 (1:24.46–1:40.10)	1:31.46 ± 19.53 (1:23.88–1:39.03)	1:21.96 ± 20.27 (1:24.10–1:39.82)	1:32.82 ± 18.46 (1:25.67–1:39.98)	[0.370]; 0.038
D4_SSP_ (85% ME)	1:29.68 ± 21.92 (1:21.18–1:38.18)	1:29.62 ± 20.32 (1:21.74–1:37.50)	1:28.51 ± 19.52 (1:20.94–1:36.80)	1:30.58 ± 19.07 (1:23.19–1:37.98)	[0.202]; 0.055
D5_SSP_ (85% ME)	1:29.05 ± 21.77 (1:20.61–1:37.49)	1:28.92 ± 20.11 (1:21.12–1:36.72)	1:28.34 ± 19.84 (1:20.65–1:36.03)	1:29.38 ± 18.82 (1:22.09–1:36.68)	[0.770]; 0.014
D6_SSP_ (90% ME)	1:28.45 ± 22.49 (1:19.73–1:37.17)	1:27.76 ± 20.83 (1:19.69–1:35.84)	1:26.05 ± 19.71 (1:18.41–1:33.70)	1:27.33 ± 19.55 (1:19.75–1:34.92)	[0.169]; 0.060
D7_SSP_ (95% ME)	1:27.09 ± 21.17 (1:18.89–1:35.30)	1:26.48 ± 20.36 (1:18.58–1:34.37)	1:24.70 ± 19.70 (1:17.06–1:32.34)	1:25.27 ± 18.77 (1:17.99–1:32.55)	**[0.035]; 0.100**
D8_SSP_ (100% ME)	1:25.64 ± 21.07 ^‡^ (1:17.47–1:33.81)	1:23.48 ± 19.07 (1:16.09–1:30.88)	1:22.57 ± 19.88 (1:14.87–1:30.28)	1:23.39 ± 18.92 (1:16.06–1:30.73)	**[0.009]; 0.132**
Total time of DSP test	11:53.72 ± 2:44.84 (10:49.80–12:57.64)	11:49.86 ± 2:34.51 (10:49.94–12:49.77)	11:45.39 ± 2:36.69 (10:44.64–12:46.15)	11:53.35 ± 2:25.74 (10:56.84–12:49.87)	[0.434]; 0.032

The results are expressed as the mean ± standard deviation and 95% confidence interval (in parentheses). Data were analyzed with ANOVA with repeated measurements (RM ANOVA); the effect size expressed as partial eta square (*η*^2^*_p_*). Results in bold refer to statistically significant differences. ^†^ T_1_ vs. T_4_: *p* = 0.003. ^‡^ T_1_ vs. T_3_: *p* = 0.007.

## Data Availability

The results of the study are presented clearly, honestly, and without fabrication, falsification, or inappropriate data manipulation. The deidentified data are available from the corresponding author upon reasonable request.
